# Targeting the conserved coronavirus octamer motif GGAAGAGC is a strategy for the development of coronavirus vaccine

**DOI:** 10.1186/s12985-023-02231-8

**Published:** 2023-11-15

**Authors:** Ching-Hung Lin, Feng-Cheng Hsieh, Yu-Chia Chang, Cheng-Yao Yang, Hsuan-Wei Hsu, Chun-Chun Yang, Hon-Man-Herman Tam, Hung-Yi Wu

**Affiliations:** 1 Graduate Institute of Veterinary Pathobiology, College of Veterinary Medicine, National Chung Hsing University, Taichung, 40227 Taiwan; 2 Department of Veterinary Medicine, College of Veterinary Medicine, National Chung Hsing University, Taichung, 40227 Taiwan

**Keywords:** Coronavirus, Coronavirus octamer, Vaccine, Translation, Replication

## Abstract

**Background:**

Coronaviruses are pathogens of humans and animals that cause widespread and costly diseases. The development of effective strategies to combat the threat of coronaviruses is therefore a top priority. The conserved coronavirus octamer motif 5’GGAAGAGC3’ exists in the 3’ untranslated region of all identified coronaviruses. In the current study, we aimed to examine whether targeting the coronavirus octamer motif GGAAGAGC is a promising approach to develop coronavirus vaccine.

**Methods:**

Plaque assays were used to determine the titers of mouse hepatitis virus (MHV)-A59 octamer mutant (MHVoctm) and wild-type (wt) MHV-A59 (MHVwt). Western blotting was used for the determination of translation efficiency of MHVoctm and MHVwt. Plaque assays and RT-qPCR were employed to examine whether MHVoctm was more sensitive to interferon treatment than MHVwt. Weight loss, clinical signs, survival rate, viral RNA detection and histopathological examination were used to evaluate whether MHVoctm was a vaccine candidate against MHVwt infection in BALB/c mice.

**Results:**

In this study, we showed that (i) the MHVoctm with mutation of coronavirus octamer was able to grow to high titers but attenuated in mice, (ii) with the reduced multiplicity of infection (MOI), the difference in gene expression between MHVoctm and MHVwt became more evident in cultured cells, (iii) MHVoctm was more sensitive to interferon treatment than MHVwt and (iv) mice inoculated with MHVoctm were protected from MHVwt infection.

**Conclusions:**

Based on the results obtained from cultured cells, it was suggested that the synergistic effects of octamer mutation, multiplicity of infection and immune response may be a mechanism explaining the distinct phenotypes of octamer-mutated coronavirus in cell culture and mice. In addition, targeting the conserved coronavirus octamer motif is a strategy for development of coronavirus vaccine. Since the conserved octamer exists in all coronaviruses, this strategy of targeting the conserved octamer motif can also be applied to other human and animal coronaviruses for the development of coronavirus vaccines, especially the emergence of novel coronaviruses such as SARS-CoV-2, saving time and cost for vaccine development and disease control.

**Supplementary Information:**

The online version contains supplementary material available at 10.1186/s12985-023-02231-8.

## Background

Coronaviruses (CoVs) are pathogens of humans and animals with medical importance that cause widespread and costly diseases. In humans, severe acute respiratory syndrome (SARS) [1], Middle East respiratory syndrome (MERS) [2] and novel viral pneumonia (officially called COVID-19) [2, 3] have posed serious public health concerns. In animals, diseases caused by porcine epidemic diarrhea virus (PEDV), infectious bronchitis virus (IBV) and bovine coronavirus (BCoV) have also led to economic losses worldwide [4–8]. The development of effective strategies to combat the threat of coronaviruses is therefore a top priority.

CoV is in the order *Nidovirales*, the family *Coronaviridae* and the subfamily *Orthocoronavirinae*, which contains four genera, *Alphacoronavirus*, *Betacoronavirus*, *Gammacoronavirus* and *Deltacoronavirus* (http://ictvonline.org/virusTaxonomy.asp). CoV contains a single-stranded, positive-sense RNA genome with a length of 26 ~ 30 kilobases (kb) [9, 10]. The genome structure comprises both 5’ and 3’ untranslated regions (UTRs), including a 5’ cap and a 3’ poly(A) tail with open reading frames (ORFs) in between [5]. RNA elements that are important for gene expression are collectively referred to as *cis*-acting RNA elements. Multiple *cis*-acting RNA elements located in the 5’ and 3’ termini of the genome have been demonstrated to be required for coronavirus gene expression [11–18]. The multiple RNA secondary structures located in the 5’ terminus of *Betacoronavirus* and *Alphacoronavirus* have been demonstrated to be required for replication [14, 15, 19, 20].

The higher-ordered structures in the 3’ UTR of *Betacoronavirus* contain the 5’-most bulged stem‒loop (BSL), hair-pin pseudoknot (PK) and 3’-most hypervariable region (HVR) [20]. The functions of the higher-ordered structures BSL and PK in replication have also been determined in *Betacoronavirus*, including BCoV and mouse hepatitis virus (MHV) [21, 22].The two *cis*-acting RNA elements BSL and PK overlap each other and therefore are structurally precluded from existing simultaneously [23, 24]. In SARS-CoV-2-infected cells, the PK is absent, and only the BSL is observed [25]. However, the formation of both BSL and PK structures has been demonstrated to be necessary for replication during infection [12, 18, 21]. Since structurally both BSL and PK cannot exist simultaneously but functionally both structures are important for coronavirus viability, it is speculated that RNA conformational switching between BSL and PK structures must occur during infection; however, the regulatory mechanism remains to be elucidated. At the 3’-most of the 3’ UTR is a higher-ordered secondary structure, but its sequence and the structure are not well conserved across the coronavirus genera. Thus, it is referred to as the hypervariable region (HVR) [23, 24]. However, a conserved sequence motif 5’GGAAGAGC3’, designated octamer (OCT) [26, 27], is identified within the HVR in all coronaviruses based on analysis of the available coronavirus sequences in GenBank [9, 11, 22, 24] including SARS-CoV-2 [10]. The conserved motif, therefore, is predicted to play a critical role in the biology of coronavirus. The previous study [22] suggests that mouse hepatitis virus (MHV)-A59, a mouse coronavirus, with a deleted HVR (including octamer motif) grows slower than wild-type (wt) MHV-A59 at an earlier stage of infection but reaches a near-wt titer at a later stage of infection in cell culture; however, in contrast to wt MHV-A59, MHV-A59 with a deleted HVR does not cause clinical signs or significant weight loss, and thus is attenuated in mice. Consequently, the authors concluded that the HVR does not function in viral RNA synthesis in tissue culture but is important for pathogenesis in mice.

In the current study, we aimed to examine whether targeting the conserved coronavirus octamer motif GGAAGAGC is a promising approach to develop coronavirus vaccine. we also aimed for seeking the possible mechanism explaining why coronavirus with octamer mutation can grow to high titers in cell culture but is attenuated in mice. In addition, based on the features of growth to high titers in cell culture but attenuation in mice, we further examined whether the octamer-mutated coronavirus has the potential as a vaccine candidate. Since the octamer exists in all coronaviruses, if coronavirus with octamer mutation is an appropriate vaccine candidate, the strategy of mutating the octamer may also be applied to other human and animal coronaviruses for vaccine development, especially for the emerging coronaviruses such as SARS-CoV-2, saving time and cost for vaccine development and disease control.

## Methods

### Cells

The mouse L (ML) cells were obtained from David A. Brian (University of Tennessee, TN). ML cells and BHK cells with MHV-A59 receptor (BHK-MHVR cells) were grown in Dulbecco’s modified Eagle’s medium (DMEM) supplemented with 10% fetal bovine serum (HyClone, UT, USA) at 37 °C with 5% CO_2_.

### Construction of wt MHV-A59 (MHVwt) and MHV-A59 with mutated octamer (MHVoctm)

The infectious clone MHV-A59-1000 (icMHV) was created and kindly provided by Dr. Ralph Baric, and the reverse-genetics system was used to construct wt MHV-A59 (MHVwt) and MHV-A59 with mutated octamer (MHVoctm) [28]. The assembled cDNA fragments were in vitro-transcribed using the T7 mMessage mMachine kit (AM1344, Thermo Fisher Scientific, Waltham, USA) and the obtained full-length viral RNA was transfected into BHK cells with MHV-A59 receptor (BHK-MHVR cells). After 48 h of transfection, the supernatant containing MHVwt or MHVoctm was collected.

### Animals

The animal study was reviewed and approved (IACUC No.: 107–145) by the Institutional Animal Care and Use Committee of National Chung Hsing University, Taiwan. Mice were maintained according to the guidelines established in the “Guide for the Care and Use of Laboratory Animals” prepared by the Committee for the Care and Use of Laboratory Animals of the Institute of Laboratory Animal Resources Commission on Life Sciences, National Research Council, USA.

### Western blotting assay

The target proteins were detected using Western blotting [29]. In brief, the cell lysates were collected from cells or mice and then quantitated using Bradford protein assay. The cell lysates were then subjected to electrophoresis with 10% SDS polyacrylamide gel. The proteins were separated and then transferred to polyvinylidene difluoride membranes. Primary antibodies against MHV-A59 N protein (provided by Dr. Paul Masters) and β actin were used followed by incubation with the corresponding secondary antibody. Enhanced chemiluminescence (ECL) was then employed to detect target proteins followed by exposure to Kodak XAR-5 film (Kodak, Rochester, NY, USA) for imaging.

### Determination of virus titer

ML cells were grown in six-well Costar plates with confluent (Costar, Cambridge, Mass., U.S.A.). Viruses MHVwt or MHVoctm collected from BHK-MHVR, ML cells or livers of mice with serial dilution was then added into ML cells. At 1 h postinfection (hpi), ML cells were washed with DMEM followed by an agarose overlay containing DMEM, 0.6% agarose and 2% FBS. The infected ML cells were then incubated for 48 h with 5% CO_2_ at 37 °C. The viral plaques were visualized by fixation with formaldehyde and staining with 0.1% crystal violet. By scoring the number of foci, the virus titer was then determined.

### Determination of the effect of the octamer on innate immunity

To evaluate the effect of the octamer on the sensitivity of IFNβ, ML cells in 2 ml of DMEM were treated with IFNβ at final concentrations of 0, 10^3^ or 10^4^ U/ml. After 16 h of treatment, IFNβ-treated ML cells were mock-infected or infected with MHVwt or MHVoctm at an MOI of 0.01. Total cellular RNA was collected at 8, 16 and 24 h post-infection.

### RT-qPCR

To quantitate the synthesis of viral RNA and cellular mRNAs for IFN, OAS and ISG15, 3 µg of TRIzol-extracted total cellular RNA was used for the RT reaction. For qPCR, SYBR® green amplification mix (Roche Applied Science, Mannheim, Germany) and oligonucleotides were used according to the manufacturer’s protocol. Dilutions of plasmids containing the same gene as the detected viral RNA or cellular mRNAs were run in parallel with the quantitated cDNA for use in standard curves (dilutions ranged from 10^8^ to 10 copies of each plasmid).

### Determination of phenotype and gene expression for MHVoctm in mice

To examine the phenotype of MHVwt and octamer mutant MHVoctm in mice, 3-week-old male BALB/c mice were used for the study. Mice were divided into 3 groups in which mice are intraperitoneally inoculated with DMEM (mock), 10^6^ pfu MHVwt (wt) or 10^6^ pfu oct mutant MHVoctm (octm). During the infection, physical examination including body weight, clinical signs and survival rate was performed [22]. To determine the gene expression, 3-week-old male BALB/c mice were also divided into 3 groups in which mice were intraperitoneally inoculated with DMEM (mock), 10^6^ pfu MHVwt (wt) or 10^6^ of pfu oct mutant MHVoctm (octm). At 1, 3 and 5 days postinfection (dpi), mice were scarified and livers were collected for histopathology examination, RT-qPCR and Western blotting.

### Histopathology examination

The collected livers were formalin-fixed and proceeded according to standard protocol followed by staining with hematoxylin and eosin (HE). Liver sections were examined under light microscopy and imagined by a digital scanner.

### Evaluation of MHVoctm as a live-attenuated vaccine candidate

To evaluate whether the octamer mutant MHVoctm can be an attenuated vaccine to protect mice from MHVwt infection, 3-week-old male BALB/c mice were divided into 4 groups in which mice were intraperitoneally inoculated with DMEM (groups DDD, DOW and DDW) or inoculated with 10^6^ pfu of mutant MHVoctm (group OOW). At 7 days postinfection, mice were intraperitoneally inoculated with DMEM (groups DDD and DDW) or infected with 10^6^ pfu of mutant MHVoctm (groups DOW and OOW). At 17 days postinfection, mice were intraperitoneally inoculated with DMEM (DDD) or challenged with 10^6^ pfu of MHVwt (groups DOW, OOW and DDW). During the inoculation and after the challenge, physical examination including body weight, clinical signs and survival rate was performed [22]. At 5 days postchallenge, mice were scarified, and livers were collected for histopathology examination, RT-qPCR and Western blotting. To test whether octamer mutant MHVoctm can be an attenuated vaccine by reducing the dosage and vaccination times, 3-week-old male BALB/c mice were used for the study. Mice were divided into 5 groups in which mice are respectively inoculated with DMEM (groups DD and DW), different pfu of oct mutant MHVoctm (10^2^ pfu for O2W; 10^4^ pfu for O4W; 10^6^ pfu for O6W). During the inoculation, physical examination including body weight, clinical signs and survival rate was performed. At 10 days postinfection, mice in group DD were inoculated with DMEM and mice in groups O2W, O4W, O6W and DW were challenged with 10^6^ pfu of MHVwt. After the challenge, physical examination including body weight, clinical score and survival rate was also performed. At 10 days postchallenge (dpc), mice were scarified, and livers were collected for histopathology examination, RT-qPCR and Western blotting.

### Serum virus neutralization assay

To test whether the octamer mutant MHVoctm can induce neutralizing antibody production, 3-week-old male BALB/c mice were used. The mice were divided into 6 groups in which mice were inoculated with DMEM (groups DD, DO2, DO4, DO6 and DW6) or 10^6^ of the octamer mutant MHVoctm (O6O6). At 10 days post-1st infection, the mice in group DD were inoculated with DMEM, and the mice in groups DO2, DO4, DO6 and O6O6 were infected with 10^2^, 10^4^, or 10^6^ or with 10^6^ pfu of oct mutant MHVoctm, respectively. The mice in group DW6 were infected with 10^6^ pfu of MHVwt. During the inoculation, physical examination of features including body weight, clinical signs and survival rate was performed. At 10 days post-2nd infection, blood samples were collected, and serum was prepared. ML cells (2 × 10^4^) were seeded in 96-well plates in DMEM supplemented with 10% fetal bovine serum (HyClone, UT, USA) and incubated at 37 °C under 5% CO_2_ for 24 h. Serial twofold dilutions starting at 1:10 in 50 µl were performed in 96-well plates. After serial dilution of serum, 50 µl of MHV (10^4^ pfu) was dispensed into wells. In the neutralization step, the mixtures of virus with serum were incubated at 37 °C with 5% CO_2_ for 1 h. After incubation, the medium was completely removed from the ML cell-seeded 96-well plate, and the neutralized mixtures were transferred to the ML cell-seeded 96-well plate. After incubation at 37 °C with 5% CO_2_ for 24 h, the monolayers were fixed with formalin (10%) and stained with crystal violet (0.1%) for measurement of neutralizing antibodies. The titers of neutralizing antibodies were calculated as the reciprocal of the highest dilution of serum showing less than 50% CPE in the cell lawn [30, 31].

## Results

### Octamer-mutated coronavirus can grow to high titers in cultured cells at different multiplicities of infections

The conserved octamer motifs are identified and found to be located 70 to 90 nucleotides upstream of the poly(A) tail in coronaviruses from different genera based on the analysis of the available coronavirus sequences in GenBank [9, 11, 22, 24]. The examples of the conserved octamers derived from different genera of coronaviruses are shown in Table S1, although few coronavirus species contain one to three nucleotides that differ from those in the conserved octamer GGAAGAGC.

**Fig. 1:**
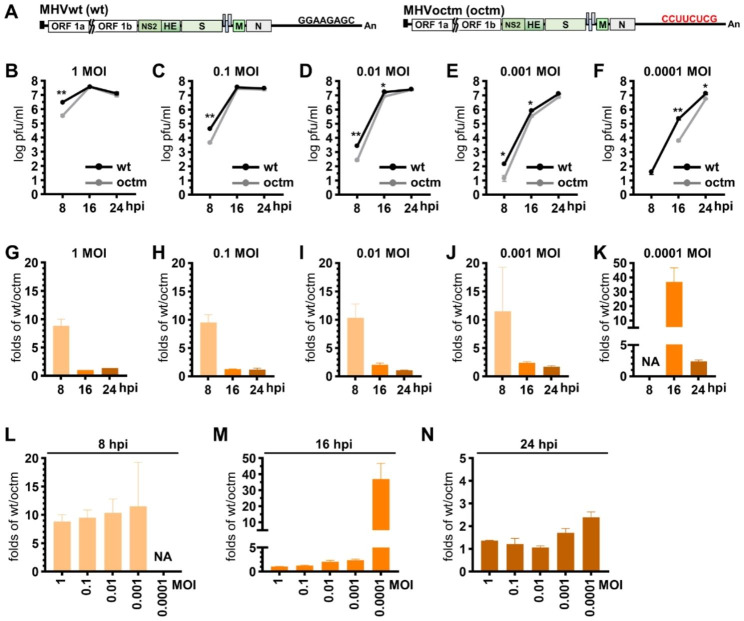
Growth kinetics of octamer-mutated coronavirus and wt coronavirus at different multiplicities of infections in cultured cells. **(A)** The genome structure of wt MHV-59 (MHVwt) and MHV-A59 with octamer mutation (MHVoctm). **(B)-(F)** Growth kinetics of MHVwt and MHVoctm in ML cells infected with MOIs of 1, 0.1, 0.01, 0.001 or 0.0001. **(G)-(K)** The folds of virus titer of MHVwt over MHVoctm (wt/octm) at different times postinfection (8,16, and 24 hpi) with MOIs of 1, 0.1, 0.01, 0.001 or 0.0001 derived from Figures (B)-(F). **(L)-(N)** The folds of virus titer of MHVwt over MHVoctm (wt/octm) with different MOIs at 8 **(L)**, 16 **(M)** and 24 **(N)** hpi derived from Figures **(B)-(F)**. The values in **(B)-(N)** represent the mean ± standard deviation (SD) of three individual experiments. Statistical significance was evaluated using a t test: * P < 0.05, ** P < 0.01. MOI, multiplicity of infection; hpi, hours postinfection; MHVwt or wt, wild-type MHV; MHVoctm or octm, MHV with octamer mutation

Based on a previous study [22], MHV-A59 with point mutation and complete deletion of the octamer motif can replicate well in cultured cells. In addition, MHV-A59 with complete deletion of the octamer motif has been demonstrated to be attenuated in mice [22]. However, whether MHV-A59 with complete mutation of the octamer motif (that is, mutation from GGAAGAGC to CCUUCUCG) can also replicate to high titers and whether such a mutation can lead to attenuation in mice remain unknown. Accordingly, MHV-A59 with complete mutation of the octamer motif was selected for the current study. To examine whether the coronavirus with complete mutation in octamer motif had the aforementioned features, wt octamer GGAAGAGC was mutated to CCUUCUCG and thus wild-type (wt) MHV-A59 (MHVwt) and MHV-A59 octamer mutant (MHVoctm) full-length cDNA (Fig. 1A) were constructed. Different multiplicities of infection (MOIs) of MHVoctm and MHVwt ranging from 1 to 0.0001 were used to infect ML cells, and supernatants were collected at different time points of infection. As shown in Fig. 1B-N, it was found that (i) at 8 hpi, with the reduced MOI from 1 to 0.0001, the virus titers for both MHVwt and MHVoctm were significantly decreased (from 3.0 × 10^6^ to 3.9 × 10^1^ for MHVwt and from 3.5 × 10^5^ to 1.4 × 10^1^ for MHVoctm), and the virus titer for MHVoctm was undetectable at an MOI of 0.0001 (Fig. 1B-F); (ii) at 8 hpi, with the reduced MOI from 1 to 0.001, the difference in virus titer between MHVwt and MHVoctm was significant (8.3- to 11.5-fold) (Fig. 1L); (iii) at 16 hpi, with the MOI reduced from 1 to 0.001, the virus titer was slightly decreased for MHVwt (from 3.9 × 10^7^ to 8.1 × 10^5^) and MHVoctm (from 3.7 × 10^7^ to 3.4 × 10^5^) (Fig. 1B-E), and the difference in virus titer between MHVwt and MHVoctm was slightly increased (from 1.0- to 2.4-fold) (Fig. 1M); however, with 0.0001 MOI, the difference was significantly increased (36.9-fold) (Fig. 1F, K and M) ; (iv) at 24 hpi, even with the reduced MOI, the virus titer for both MHVwt and MHVoctm was high and similar (Fig. 1B-F), and the difference was only slightly increased (from 1.1 to 2.4 folds) (Fig. 1N). Therefore, with a reduction in MOI from 1 to 0.0001, the replication efficiency for both MHVwt and MHVoctm was significantly decreased during the earlier stage of infection (especially at 8 hpi). In addition, the difference between MHVwt and MHVoctm in virus titer during the earlier stage of infection (8 and 16 hpi) was increased. Notably, with 0.0001 MOI, the virus titer for MHVoctm was even undetectable at 8 hpi and the difference was significantly increased (36.9-fold) at 16 hpi. However, the virus titer for both MHVwt and MHVoctm was high and similar at the later stage of infection (24 hpi). The results suggest that, with reduced MOI for infection, the difference between MHVwt and MHVoctm in virus titer at the earlier stage of infection (8 and 16 hpi) become evident; however, even with mutated octamer and reduced MOI for infection, MHVoctm still can grow to high titer similar to MHVwt in cultured cells at the later stage of infection, establishing MHVoctm as a potential candidate for the vaccine development.

### The coronavirus with mutated octamer leads to the reduced and lagged synthesis of viral proteins

**Fig. 2:**
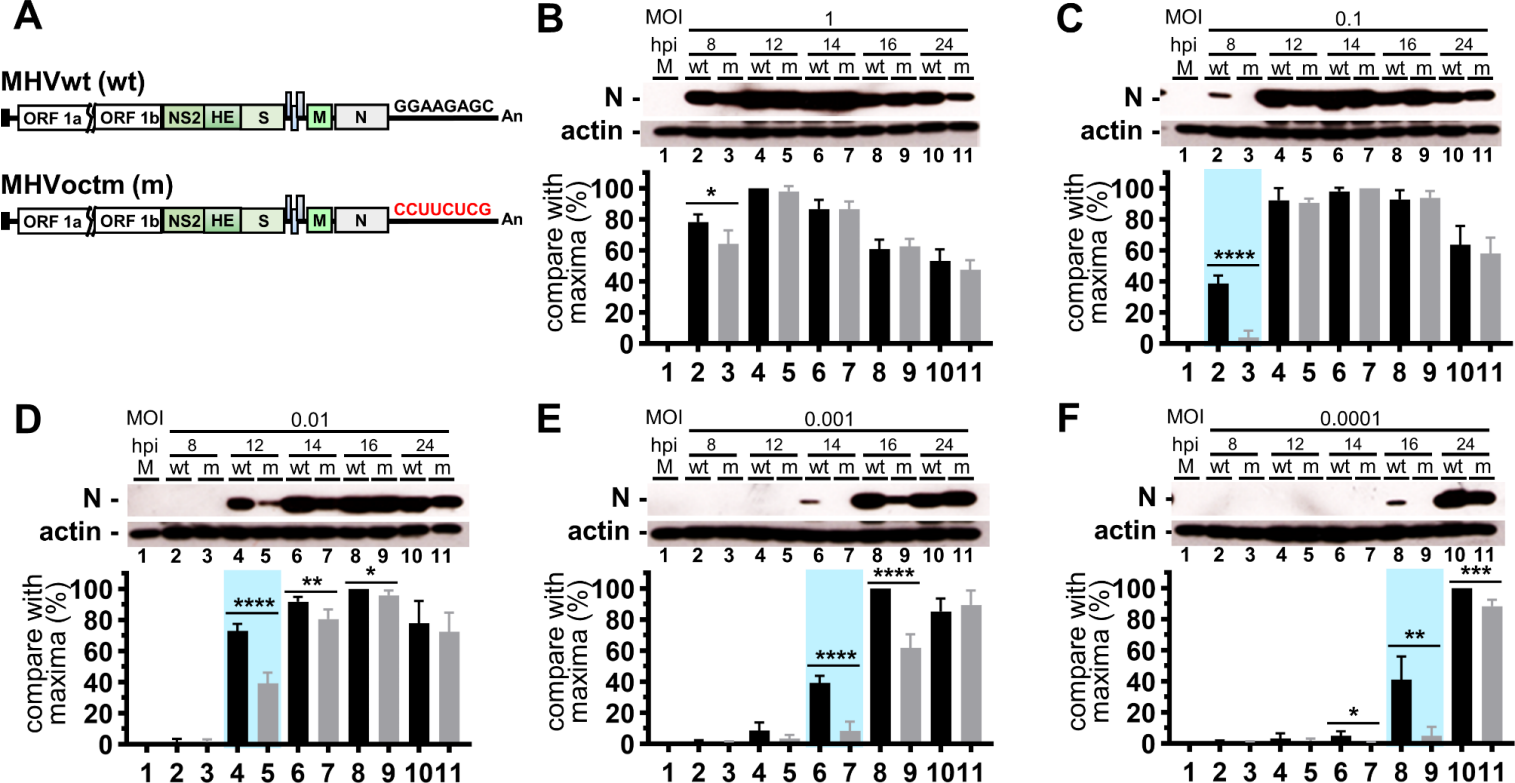
The coronavirus with mutated octamer leads to the reduced and lagged synthesis of viral proteins. **(A)** The genome structure of wt MHV-59 (MHVwt) and MHV-A59 with octamer mutation (MHVoctm). **(B)-(F)** Detection and comparison of N protein synthesis between MHVwt and MHVoctmin in ML cells infected with different MOIs (1, 0.1, 0.01, 0.001 and 0.0001, **B-F)** at different times postinfection by Western blotting. The amounts of the N protein were normalized to those of actin. The values in **(B)-(F)** represent the mean ± standard deviation (SD) of three individual experiments. Statistical significance was evaluated using a t test: * P < 0.05, ** P < 0.01, **** P < 0.0001. MOI, multiplicity of infection; M, mock infection; N, nucleocapsid protein; hpi, hours postinfection; MHVwt or wt, wild-type MHV; MHVoctm or m, MHV with octamer mutation

Since (i) MOI can affect the replication difference between octamer-mutated (MHVoctm) and wild-type (MHVwt) coronavirus during infection (Fig. 1), (ii) the mice may be infected with much lower MOI of virus than cultured cells and (iii) the efficiency of viral proteins synthesis is also a factor affecting the pathogenicity, it is hypothesized that, with the reduced MOI, the decreased translation efficiency of MHVoctm in comparison with that of MHVwt at the earlier stage of infection may suggest its potential to be attenuated in mice, supporting its role as a vaccine candidate. To examine the hypothesis, different MOIs of MHVoctm and MHVwt ranging from 1 to 0.0001 were used to infect ML cells, and cell lysates were collected at different time points of infection. As shown in Fig. 2B, with a higher MOI of 1, in comparison with MHVwt, MHVoctm showed reduced translation efficiency at 8 h postinfection (hpi), but had almost the same translation efficiency at 12, 14, 16 and 24 hpi. With a reduction in MOI from 0.1 to 0.0001, the synthesis of detectable viral proteins was delayed (Fig. 2C-F). In addition, the translation difference between MHVoctm and MHVwt was observed, and the occurrence of the difference was also delayed (Fig. 2C-F). Consequently, the results suggest that, with reduced MOI for infection in cultured cells (Fig. 2B-F), (i) the synthesis of detectable viral proteins gradually lagged after infection and (ii) the difference in translation efficiency between MHVwt and MHVoctm became significant. Since translation occurs prior to replication, the difference in virus titer with different MOI at the earlier stage of infection (Fig. 1) may be at least partly due to the effect of the octamer mutation on translation efficiency. Consequently, the results further suggest that the octamer-mutated MHVoctm has the potential to be attenuated in mice and thus is a potential vaccine candidate.

### The coronavirus with mutated octamer is more sensitive to interferon treatment than wt coronavirus

**Fig. 3:**
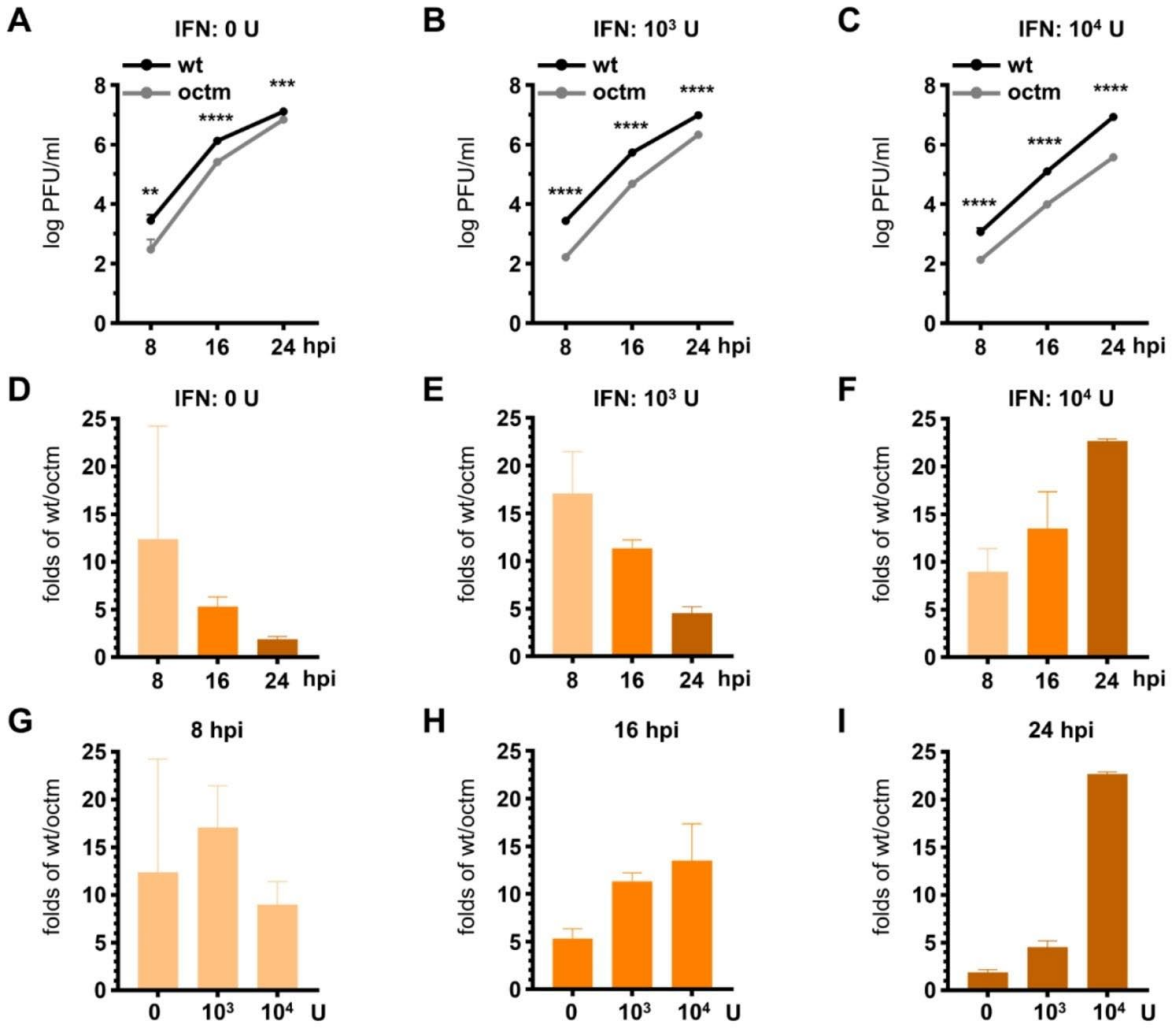
Octamer-mutated MHVoctm is more sensitive to interferon treatment than wt coronavirus MHVwt. **(A)-(C)** The growth kinetics of wt MHV-59 (MHVwt) and MHV-A59 with octamer mutation (MHVoctm) at 0.01 MOI with 0 **(A)**, 10^3^**(B)** or 10^4^**(C)** U/ml of IFNβ. **(D)-(F)** The folds of virus titer of MHVwt over MHVoctm (wt/octm) at different times postinfection (8,16, and 24 hpi) with different amounts of IFNβ (0 U/ml (D), 10^3^ U/ml **(E)** and 10^4^ U/ml **(F)**) derived from Figures **(A)-(C)**. **(G)-(I)** The folds of virus titer of MHVwt over MHVoctm (wt/octm) with different amounts of IFNβ (0, 10^3^ and 10^4^ U/ml) at 8 **(G)**, 16 **(H)** and 24 **(I)** hpi derived from Figures **(A)-(C)**. wt, wild-type MHV-A59; octm, MHV-A59 with octamer mutation; hpi, hours postinfection. Statistical significance was evaluated using a t test: ** P < 0.01, *** P < 0.001, **** P < 0.0001

It has been demonstrated that coronavirus-encoded protein can antagonize host innate immunity [32]. Consequently, since MHVoctm with mutated octamer displays less translation efficiency than MHVwt (Fig. 2), it was hypothesized that the reduced translation efficiency for octamer-mutated MHVoctm may have a weaker ability to weather the challenge of innate immunity and thus reduce the replication efficiency, which would also be a potential factor leading to the attenuated phenotype of octamer-mutated MHVoctm in vivo. To compare the capability against the challenge of innate immunity between MHVoctm and MHVwt, ML cells were first treated with different doses of interferon (IFN) β followed by infection with MHVwt or MHVoctm at an MOI of 0.01. As shown in Fig. 3A and summarized in Fig. 3D, without IFNβ treatment, the virus titer of MHVwt was higher than that of MHVoctm at 8 and 16 hpi but was similar to that of MHVoctm at 24 hpi. However, with the treatment of 10^4^ U/ml IFNβ, the difference in virus titer between MHVwt and MHVoctm was increased with time (Fig. 3C and summarized in Fig. 3F). In addition, with increased amounts (from 0, 10^3^ to 10^4^ U/ml) of IFNβ treatment, the difference in virus titer between MHVwt and MHVoctm was also increased at 16 and 24 hpi (Fig. 3H and I). The results together suggest that MHVoctm is more sensitive to IFNβ treatment than MHVwt.

**Fig. 4:**
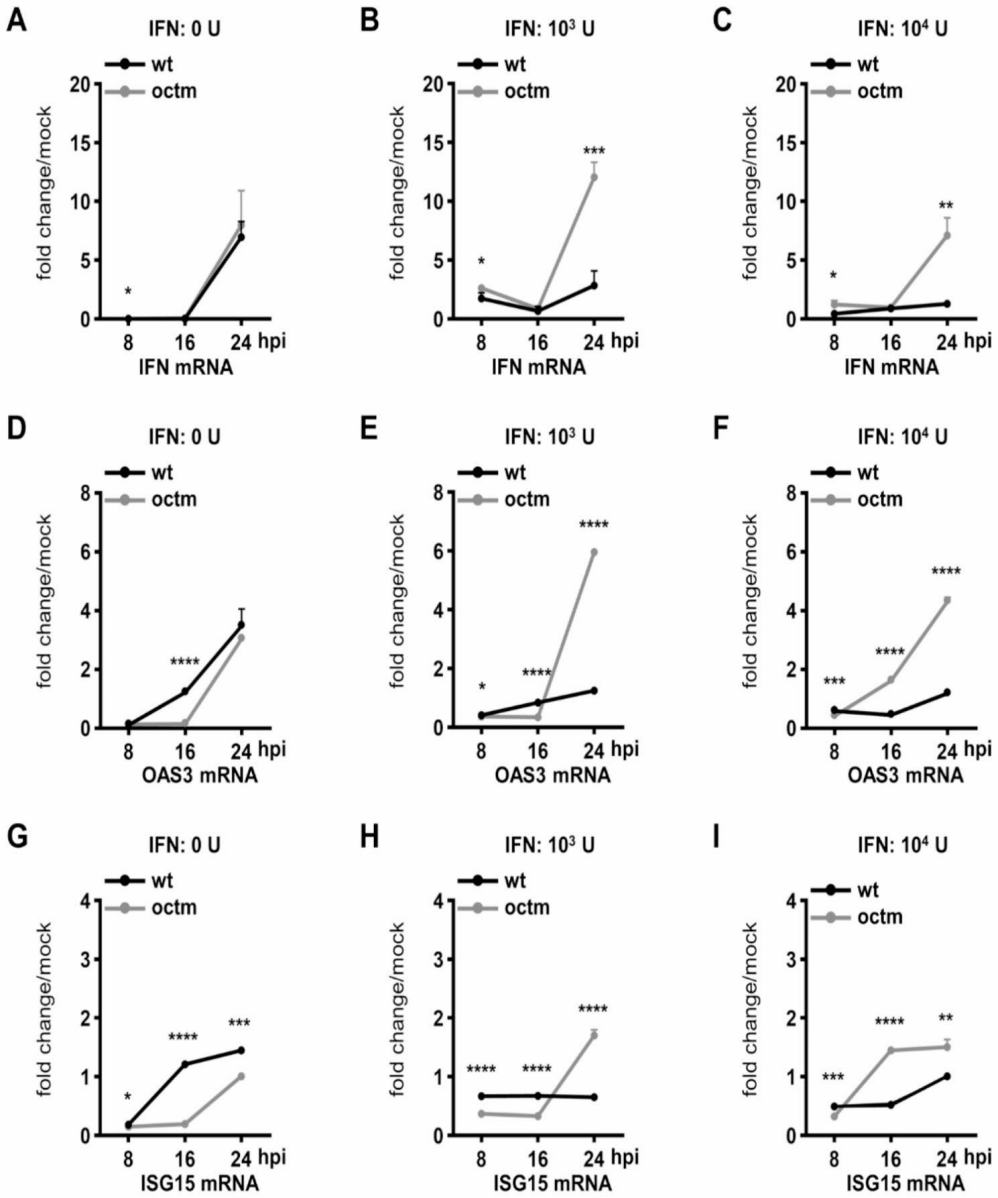
The capability of inhibiting the IFNβ signaling pathway by MHVwt and MHVoctm. **(A)-(I)** Relative amounts of IFNβ **(A)-(C)**, OAS **(D)-(F)** and ISG15 **(G)-(I)** mRNA synthesis in ML cells treated with different amounts of IFNβ followed by mock infection or infection with MHVwt or MHVoctm (0.01 MOI) at different times postinfection. “Mock” on the y-axis indicates the amount of mRNA detected from IFNβ-treated and mock-infected cells. The fold change/mock on the y-axis is presented as relative units of mRNA compared to the amount of mRNA in IFNβ-treated and mock-infected cells (the amount of mRNA in IFNβ-treated and mock-infected cells = 1). The values in **(A)-(I)** represent the mean ± standard deviation (SD) of three individual experiments. Statistical significance was evaluated using a t test: * P < 0.05, ** P < 0.01, *** P < 0.001, **** P < 0.0001. hpi, hours postinfection; IFN, interferon; OAS, 2 ′,5′-oligoadenylate synthetase; ISG15, interferon-stimulated gene 15; MHVwt or wt, wild-type MHV; MHVoctm or octm, MHV with octamer mutation

Further examination revealed that, with IFNβ treatment, the capability of inhibiting the IFNβ signaling pathway for MHVwt was overall stronger than that for MHVoctm because the mRNA levels of IFNβ (Fig. 4A-C), 2′,5′-oligoadenylate synthetase (OAS) (Fig. 4D-F) and interferon-stimulated gene 15 (ISG15) (Fig. 4G-I) in IFNβ-treated and MHVwt-infected ML cells were overall lower than those in IFNβ-treated and MHVoctm-infected ML cells. Taken together, the results suggest that MHVoctm is more sensitive to IFNβ treatment than MHVwt. Since coronavirus-encoded proteins can antagonize innate immunity [32], the weaker capability of MHVoctm to inhibit innate immunity may also be a potential factor leading to the attenuated phenotype of octamer-mutated MHVoctm in mice.

### Coronavirus with octamer mutation is attenuated in mice

**Fig. 5:**
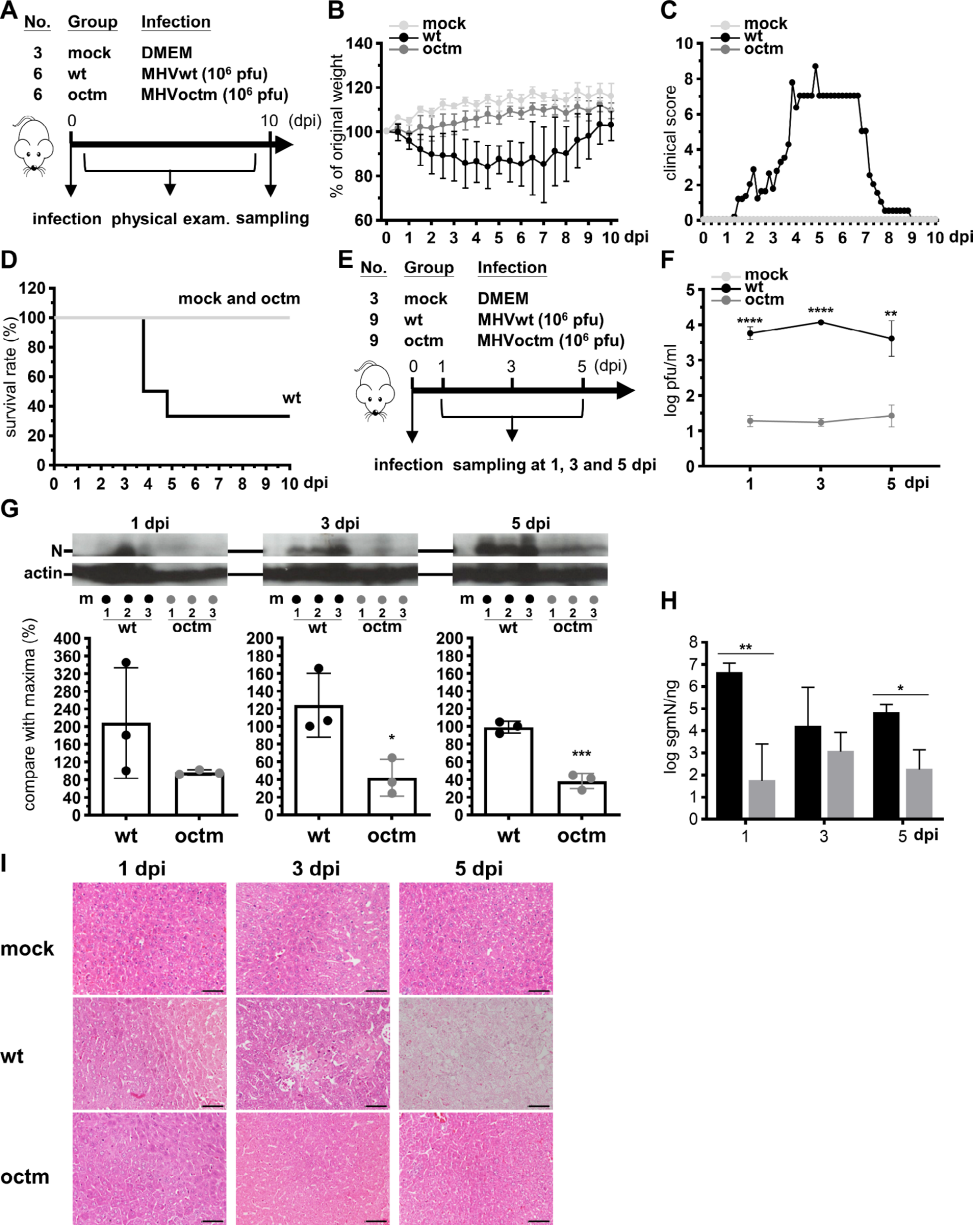
MHVoctm with octamer mutation is attenuated in mice. **(A)** Timeline for physical examination of the phenotype of MHVwt- or MHVoctm-infected mice, including body weight **(B)**, clinical score **(C)** and survival rate **(D)**. 10^6^ pfu MHVwt or MHVoctm were used for the experiment. **(E)** Timeline for physical examination of virus titer **(F)**, translation efficiency **(G)**, synthesis of viral RNA **(H)** and histopathology **(I)** in MHVwt- or MHVoctm-infected mice. Histopathology examination (scale bar = 100 μm) revealed coagulation necrosis and vacuolated hepatocytes in the livers of mice inoculated with MHVwt but not with MHVoctm. The uncropped gels for **(G)** were shown in Figure S2. dpi, days postinfection; MHVwt or wt, wild-type MHV; MHVoctm or octm, MHV with octamer mutation. Statistical significance was evaluated using a t test: ** P < 0.01, *** P < 0.001, **** P < 0.0001

Based on the results shown in Figs. 1 and 2, and 3, the octamer-mutated MHVoctm has the potential to be a vaccine candidate. Therefore, to verify whether the octamer-mutated MHVoctm can be attenuated in mice, 3-week-old male BALB/c mice were intraperitoneally inoculated with Dulbecco’s Modified Eagle Medium (DMEM), 10^6^ pfu of MHVwt or MHVoctm (Fig. 5A). Substantial weight loss, clinical signs and death were not observed in mice inoculated with MHVoctm but MHVwt (Fig. 5B-D). The levels of viral titers, proteins and RNA were also lower in MHVoctm-infected mice than in MHVwt-infected mice (Fig. 5F-H). In addition, the results shown in Fig. 5G demonstrated that MHVoctm could express viral N proteins, although the amounts of the proteins expressed by MHVoctm were much lower than those expressed by MHVwt. Thus, the results suggest that MHVoctm can replicate in mice. Histopathological examination revealed coagulation necrosis and vacuolated hepatocytes (ballooning degeneration) in the livers of mice inoculated with MHVwt, but no histopathology changes were observed in mice inoculated with MHVoctm (Fig. 5I). Together with the results obtained from cell cultures shown in Figs. 1, 2 and 3, the findings indicate that the dramatically decreased gene expression of MHVoctm in mice may be at least partly attributable to the mutated octamer and the reduced MOI, which led to decreased gene expression and thus a decreased capability to antagonize innate immunity, ultimately leading to the attenuated phenotype of the octamer mutant MHVoctm in mice. Consequently, because (i) the levels of viral titers and RNA were not decreased with the time (Fig. 5F and H) and (ii) MHVoctm could express viral protein in the livers of mice, the results suggest that MHVoctm can replicate in mice (Fig. 5G). In addition, because the levels of viral titers, proteins and viral RNA were also lower in MHVoctm-infected mice than in MHVwt-infected mice, the results suggest that MHVoctm is attenuated in mice. Since MHVoctm with the mutated octamer can replicate well in cell cultures (Fig. 1) but is attenuated in mice (Fig. 5), the results together suggest that the octamer-mutated MHVoctm is a potential vaccine candidate.

### MHVoctm can be used as an attenuated vaccine to protect mice against MHVwt infection

**Fig. 6:**
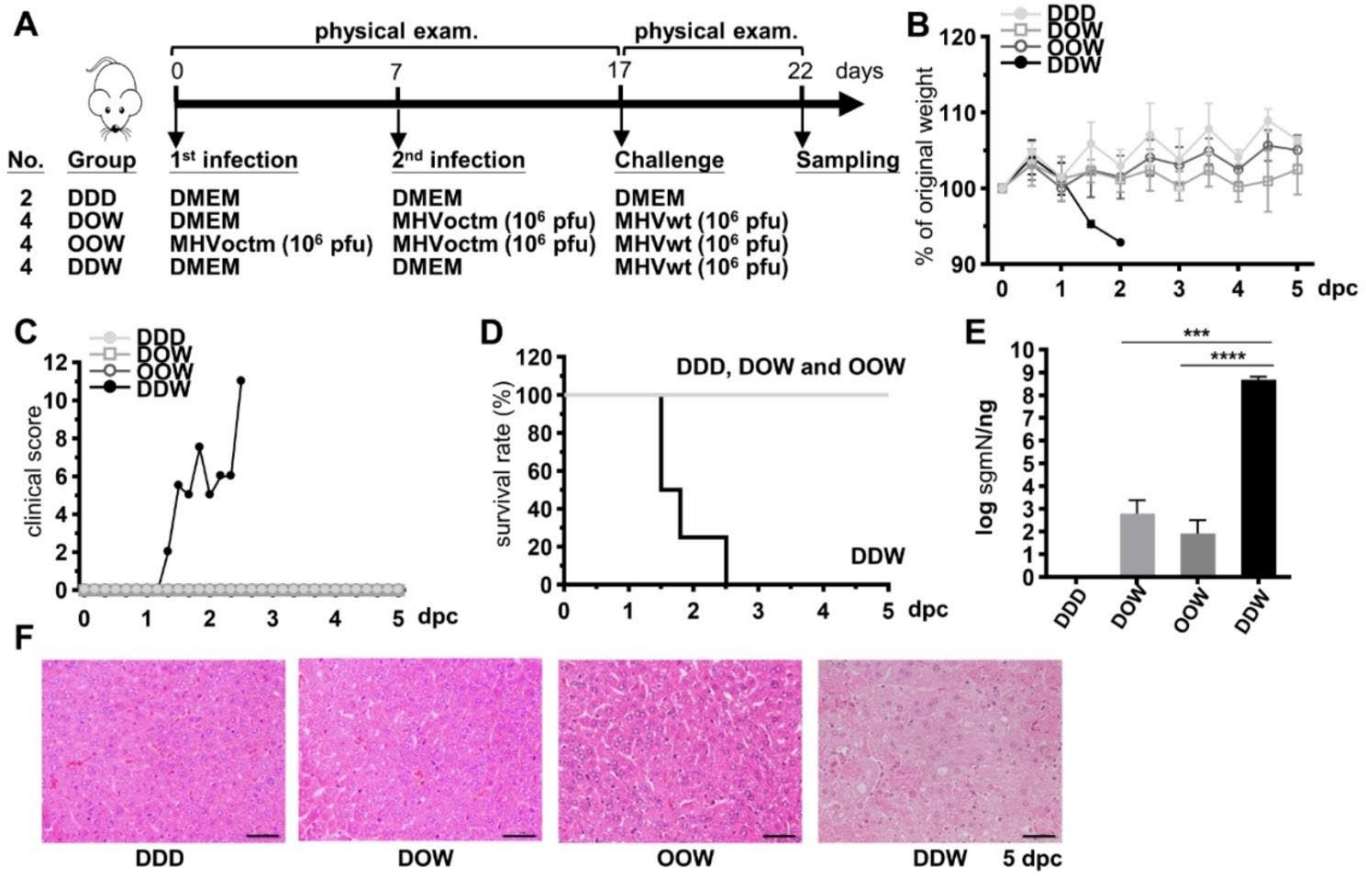
Examination of the safety and efficacy of the attenuated vaccine candidate MHVoctm. **(A)** Timeline and experimental groups for examination of the safety and efficacy of the attenuated vaccine MHVoctm. A total of 10^6^ pfu of MHVwt or MHVoctm were used for the experiment. **(B)-(F)** Examination of body weight **(B)**, clinical score **(C)**, survival rate **(D)**, synthesis of viral RNA **(E)** and pathological changes in which coagulation necrosis and vacuolated hepatocytes (ballooning degeneration) were observed in the livers of mice inoculated with DMEM wt but not with MHVoctm after challenge with MHVwt (scale bar = 100 μm) **(F)**. dpc, days postchallenge; MHVwt, wild-type MHV; MHVoctm, MHV with octamer mutation. Statistical significance was evaluated using a t test: *** P < 0.001, **** P < 0.0001

Since the octamer mutant MHVoctm can grow to high titers in cultured cells and is attenuated in mice (Figs. 1 and 5), the octamer mutant MHVoctm has the great potential as a vaccine candidate against MHVwt infection. For this purpose, 3-week-old male BALB/c mice were inoculated with DMEM or 10^6^ pfu of MHVoctm once or twice followed by challenge with 10^6^ pfu of MHVwt (Fig. 6A). Before the challenge with MHVwt, significant weight loss, clinical signs and death were not observed in all groups. After the challenge with MHVwt, weight loss, clinical signs and death were also not observed in mice inoculated with octamer mutant MHVoctm once or twice (Fig. 6B-D). In contrast, mice inoculated with DMEM started to show significant weight loss and clinical signs after 1 day of challenge and started to die after 2 days of challenge (Fig. 6B-D). In addition, the viral RNA detected from mice inoculated with octamer mutant MHVoctm once or twice was significantly less than that inoculated with DMEM (8.2 × 10^1^/ng vs. 4.3 × 10^8^/ng, Fig. 6E). By histopathological examination, coagulation necrosis and vacuolated hepatocytes (ballooning degeneration) were observed in the livers of mice inoculated with DMEM followed by challenge with MHVwt (DDW group) but not in groups of mice inoculated with octamer mutant MHVoctm once (DOW group) or twice (OOW group) followed by challenge with MHVwt (Fig. 6F). Based on the results of weight loss, clinical signs, survival rate, viral RNA detection and histopathological examination, manipulation of the conserved octamer is a promising strategy for vaccine design against coronavirus infection.

### Refinement of the vaccination strategy for the attenuated vaccine candidate MHVoctm by reducing the dosage and times of vaccination

**Fig. 7:**
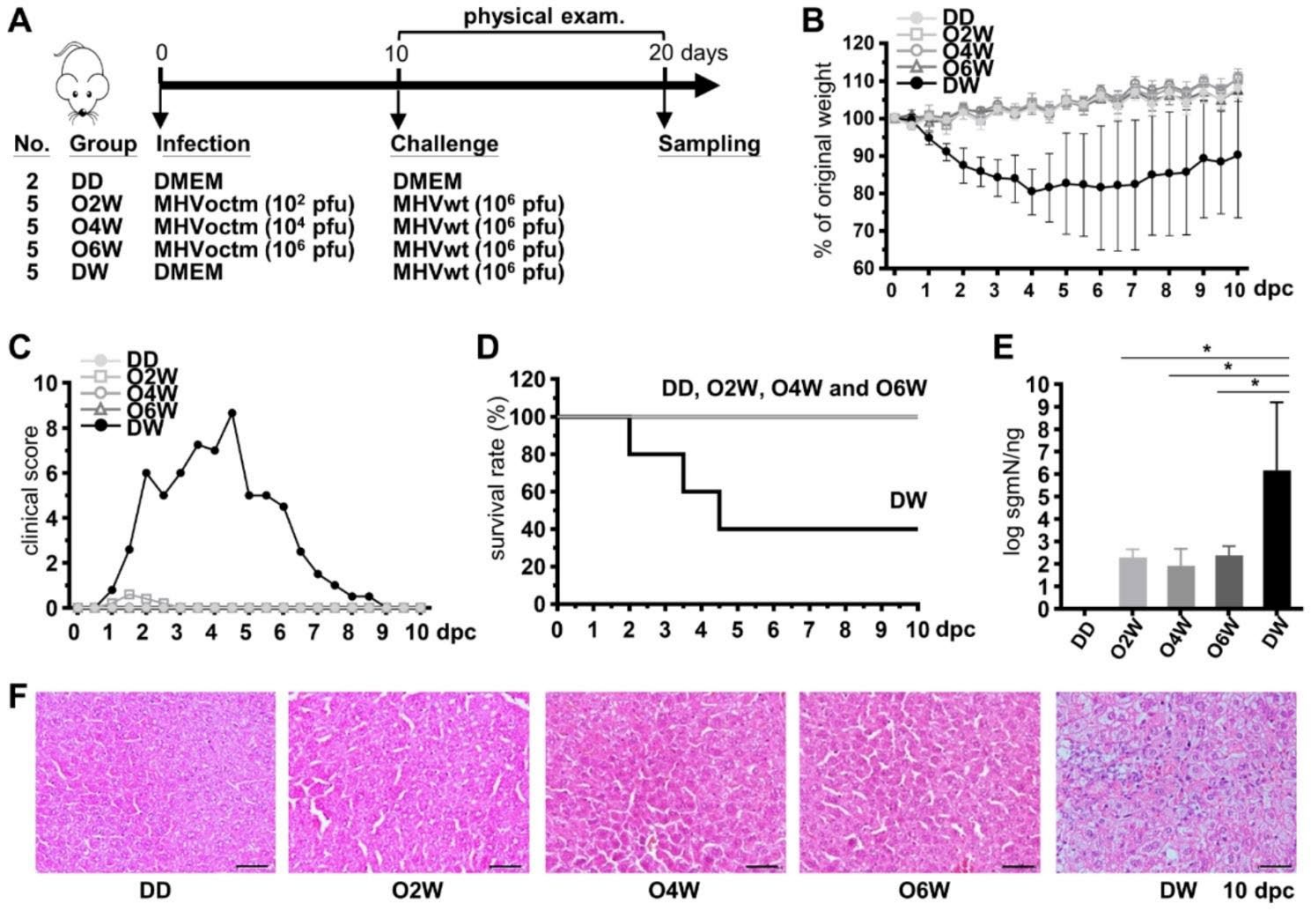
The safety and efficacy of the attenuated vaccine candidate MHVoctm with modified vaccination strategy. **(A)** Timeline and experimental groups for examination of the efficacy and safety of the attenuated vaccine MHVoctm. The reduced dosages (10^6^, 10^4^ and 10^2^ pfu) of MHVoctm and one vaccination were used for the experiment. **(B)-(F)** Examination of body weight **(B)**, clinical score **(C)**, survival rate **(D)**, synthesis of viral RNA **(E)** and pathological changes in which coagulation necrosis and vacuolated hepatocytes (ballooning degeneration) were observed in the livers of mice inoculated with DMEM but not with MHVoctm after challenge with MHVwt (scale bar = 100 μm) **(F)**. dpc, days postchallenge; MHVwt, wild-type MHV; MHVoctm, MHV with octamer mutation. Statistical significance was evaluated using a t test: * P < 0.05

To further determine the vaccine potential of the mutant MHVoctm, the vaccination strategy was refined by reducing the times and the virus dosage for vaccination. For this purpose, mice were inoculated with the octamer mutant MHVoctm only once, with 10^2^, 10^4^ or 10^6^ pfu of virus (Fig. 7A). As shown in Fig. 7B-D and F, no significant weight loss, clinical signs, death or histopathological changes were observed in mice inoculated with 10^2^, 10^4^ or 10^6^ pfu of octamer mutant MHVoctm followed by challenge with MHVwt. In addition, a much lower amount of viral RNA was detected in mice inoculated with 10^2^, 10^4^ or 10^6^ pfu of octamer mutant MHVoctm followed by challenge with MHVwt in comparison with that in mice inoculated with DMEM followed by challenge with MHVwt. (8.1 × 10^1^/ng vs. 1.5 × 10^6^/ng, Fig. 7E).

**Fig. 8:**
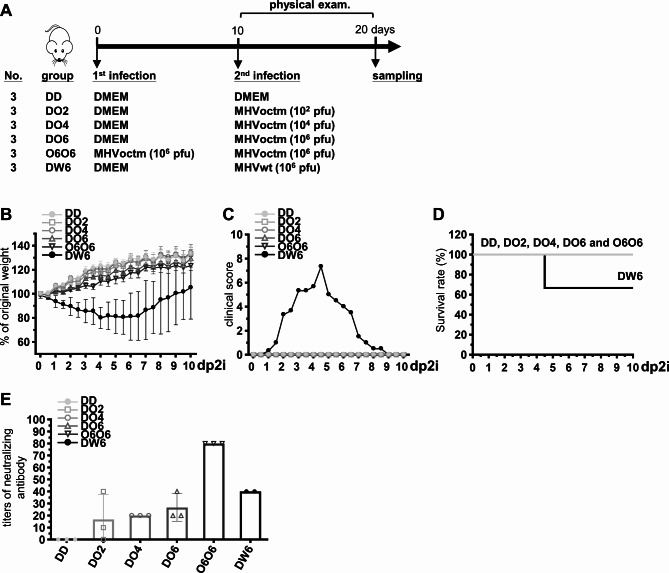
Titers of serum virus neutralization antibodies in vaccinated mice. **(A)** Timeline and experimental groups for the measurement of neutralizing antibodies. **(B)-(D)** Examination of body weight **(B)**, clinical score **(C)** and survival rate **(D)**. **(E)** Titers of neutralizing antibodies. dp2i, days post-2nd infection

To examine whether MHVoctm was able to elicit neutralizing antibody production, a serum virus neutralization assay was performed. As shown in Fig. 8 and S3, neutralizing antibodies were measured, and the average titer increased moderately in a dose-dependent manner. In addition, booster vaccination induced more neutralizing antibody production than only one vaccination. The results suggest that the octamer mutant MHVoctm can induce neutralizing antibody production.

Taken together, the results suggest that inoculating mice with 10^2^ pfu of the octamer mutant MHVoctm one time is sufficient to provide protection against MHVwt infection, thus further demonstrating that manipulation of conserved octamers is a novel and promising strategy to develop a live attenuated vaccine against coronavirus infection.

## Discussion

In this study, it is suggested that the difference in replication and translation efficiency between mutated MHV-A59 (MHVoctm) and wild-type MHV-A59 (MHVwt) becomes more evident at the earlier stage of infection with the reduced MOI in cultured cells (Figs. 1 and 2). In addition, the efficiency of both replication and translation is also much lower in MHVoctm-infected than in MHVwt-infected mice (Fig. 5). Because translation occurs prior to replication and virus titers are almost similar at the later infection in cultured cells, the difference in virus titer at the earlier stage of infection between MHVwt and MHVoctm treated with different MOIs or different doses of IFNβ (Figs. 1 and 3) therefore may be partly attributed to the reduced translation efficiency caused by octamer mutation. In addition, because coronavirus-encoded protein can antagonize host innate immunity [32], the reduced protein synthesis may also explain why (i) MHVoctm is more sensitive to treatment with IFNβ than MHVwt in cell culture, (ii) the difference in virus titer between MHVwt and MHVoctm becomes evident with the increased amounts of IFNβ in cell culture (Fig. 3) and (iii) mice infected with MHVoctm show almost no weight loss, clinical signs and histopathology (Figs. 5, 6 and 7).

Based on the results obtained from the current study, the increased difference in virus titer between MHVwt and MHVoctm may be due to the mutated octamer, reduced MOI and increased IFNβ (Figs. 1 and 3). It is also known that the octamer can affect the synthesis of coronaviral proteins (Fig. 2). In addition, with the same number of viruses for infection, mice may be infected with much lower MOI than cell culture, and thus the coronaviral protein concentration within the same number of cells in mice may be much less than that in cultured cells. Consequently, because coronavirus-encoded protein can antagonize host innate immunity [32], the octamer mutation, which leads to the reduced gene expression, may play a rate-limiting role in mice which are infected with much lower MOI than cultured cells and can launch host immune responses. Thus, it is reasoned that upon infection of wt MHV-A59 (MHVwt), MHVwt with wt octamer can synthesize viral proteins efficiently in mice and thus can overcome the defense of the host immunity, leading to high virus titer and thus the death of mice (Fig. 5). In contrast, the reduced synthesis of viral protein in mice infected with octamer-mutated MHVoctm is a rate-limiting step due to the effect of mutated octamer. The reduced and lagged synthesis of viral proteins during this rate-limiting step can further dampens the capability of the octamer-mutated MHVoctm to defend the host immune responses, resulting in further decreased virus replication, translation and subsequent pathogenicity. Accordingly, it is argued that the synergistic effects of octamer mutation, reduced MOI, and the challenge of host immunity during virus replication cycle may be the reasons leading to the distinct phenotype between the two viruses MHVwt and MHVoctm in mice (Fig. 5). The argument thus also can explain why MHVoctm with octamer mutation can grow to high virus titer in cell culture (Fig. 1) but is attenuated in mice (Fig. 5) in the current study.

An attenuated virus vaccine needs to meet the criteria of (i) growth to high titer in a suitable system, (ii) attenuation in the host and (iii) protection of the host from wild-type virus infection. Consequently, the results shown in Figs. 1, 5, 6 and 7 demonstrate that the octamer mutant MHVoctm is a good attenuated virus vaccine candidate because (i) it can be robustly produced in cell culture with a high virus titer (Fig. 1); (ii) it can replicate in mice, but the inoculated mice show no clinical signs and histopathological changes (Fig. 5); and (iii) it can protect mice from MHVwt infection (Figs. 6 and 7). Accordingly, using an octamer mutant as an attenuated vaccine has the following merits. First, because octamers are conserved in all known coronaviruses, the strategy designed in this study by mutation of the octamer can be used as a platform to immediately develop efficient vaccines once novel coronaviruses or their variants with medical importance emerge, saving time and cost. Second, because the octamer mutant can grow to high titers in cell culture (Fig. 1) and inoculation of a small number of viruses (Fig. 7) can exert a protective effect, the cost of the developed attenuated vaccine can be reduced. Third, based on the animal trial in mice (Fig. 7), only one vaccination is sufficient to provide protection against MHVwt infection, also saving time and cost. Finally, the viral RNA of MHVoctm is not detectable in respiratory secretions, stool or urine specimens during the vaccination of mice. In addition, the reversion of the octamer mutation in MHVoctm does not occur after 6 virus passages in cultured cells using the inoculum obtained from the supernatant of MHVoctm-infected cells or from livers of vaccinated mice (Lin et al., unpublished data). The results suggest that the vaccine candidate obtained by mutation of the octamer motif is stable. Thus, the vaccine candidate has the advantage of reducing the risk of virus reversion and thus increasing safety. Alternatively, since the octamer mutant can grow to a high titer, it can also be used to produce inactivated vaccines, reducing the cost and increasing the safety of vaccines. In addition, modification of the octamer sequence via point mutation or deletion, and alteration of the structure where the octamer resides to increase the replication efficiency in cell culture without causing any harmful effects in vivo are also means to refine the vaccine, and the modifications can be tested in future studies.

Because mutation of the conserved octamer can function in reducing gene expression and increasing sensitivity to innate immunity, understanding the detailed mechanisms of how the octamer functions in gene expression, and blocking the function of the octamer may be an alternative strategy to inhibit the gene expression of all coronaviruses. Thus, targeting the octamer or proteins interacting with and thus blocking the function of the octamer may also be a promising strategy to develop antivirals. Such antivirals, if developed, may have advantages over other antivirals because they can target the conserved octamer and thus exhibiting a broad-spectrum effect on all known coronaviruses. Accordingly, although we hope this will not occur, if another novel coronavirus emerges, such antivirals can immediately be applied to coronavirus-infected patients, saving time to develop new antivirals and efficiently reducing the severity of the emerging disease.

In conclusion, since the octamer exists in all coronaviruses, it could be the Achilles heel of coronaviruses because (i) the coronavirus with octamer mutation is an appropriate vaccine candidate, and this strategy of mutating the octamer may also be applied to other human and animal coronaviruses for the development of vaccines, especially the emergence of novel coronaviruses such as SARS-CoV-2, saving time and cost for disease control, and (ii) targeting the conserved octamer or proteins interacting with to block its function may also be a promising strategy to develop antivirals with a broad spectrum for the inhibition of all known coronaviruses.

### Electronic supplementary material

Below is the link to the electronic supplementary material.

Supplementary Material 1

## Data Availability

Not applicable.

## Abbreviations

MOI: Multiplicity of infection
PFU: Plaque-forming unit
CoV: Coronavirus
SARS-CoV-2: Severe acute respiratory syndrome coronavirus 2
UTR: Untranslated region
ORF: Open reading frame
IBV: Infectious bronchitis virus
PEDV: Porcine epidemic diarrhea virus
MHV: Mouse hepatitis virus
BCoV: Bovine coronavirus
ML cells: Mouse L cells
DMEM: Dulbecco’s modified Eagle’s medium
icMHV: Infectious clone MHV-A59-1000
