# Supplementary material for: Targeting the conserved coronavirus octamer motif GGAAGAGC is a strategy for the development of coronavirus vaccine
Source: Virol J. 2023 Nov 15;20:267. doi: 10.1186/s12985-023-02231-8 (PMC10652495; doi:10.1186/s12985-023-02231-8)
Supplement: Supplementary file 1 — Supplementary Material 1 [file 12985_2023_2231_MOESM1_ESM.docx]

**Supplementary Materials**

**Targeting the conserved coronavirus octamer motif GGAAGAGC is a strategy for the development of coronavirus vaccine**

Ching-Hung Lin^1^, Feng-Cheng Hsieh^1^, Yu-Chia Chang^1^, Cheng-Yao Yang^1^, Hsuan-Wei Hsu^1^, Chun-Chun Yang^1^, Hon-Man-Herman Tam^2^, Hung-Yi Wu^1*^

**1** Graduate Institute of Veterinary Pathobiology, College of Veterinary Medicine, National Chung Hsing University, Taichung 40227, Taiwan

**2** Department of Veterinary Medicine, College of Veterinary Medicine, National Chung Hsing University, Taichung 40227, Taiwan

*Corresponding Author

Corresponding Footnote: Graduate Institute of Veterinary Pathobiology, College of Veterinary Medicine, National Chung Hsing University, Taichung 40227, Taiwan

Telephone: 886-4-22840369; Fax: 886-4-22862073

Email: hwu2@dragon.nchu.edu.tw


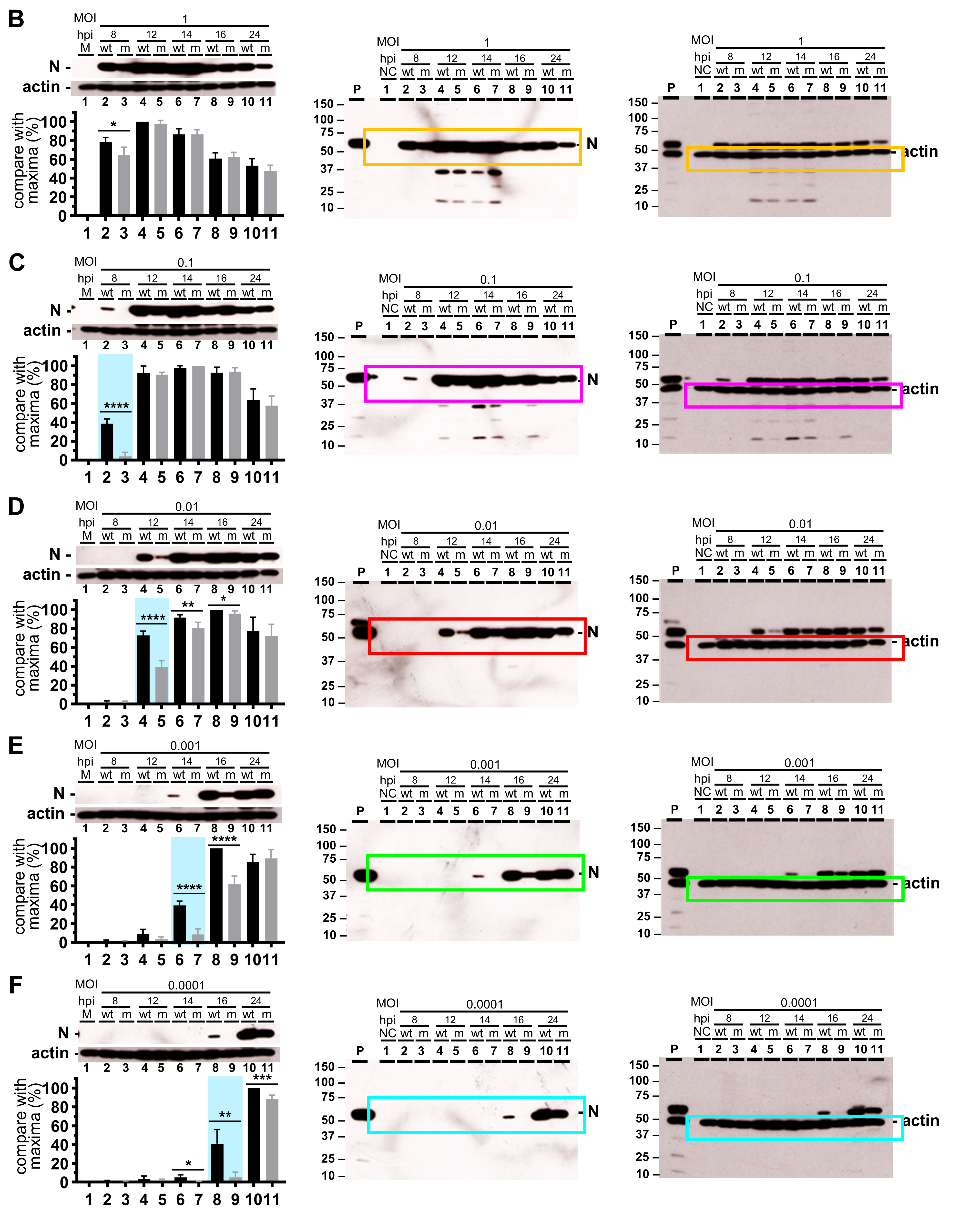


**Figure S1. Uncropped gels for Figures 2B-2F.**


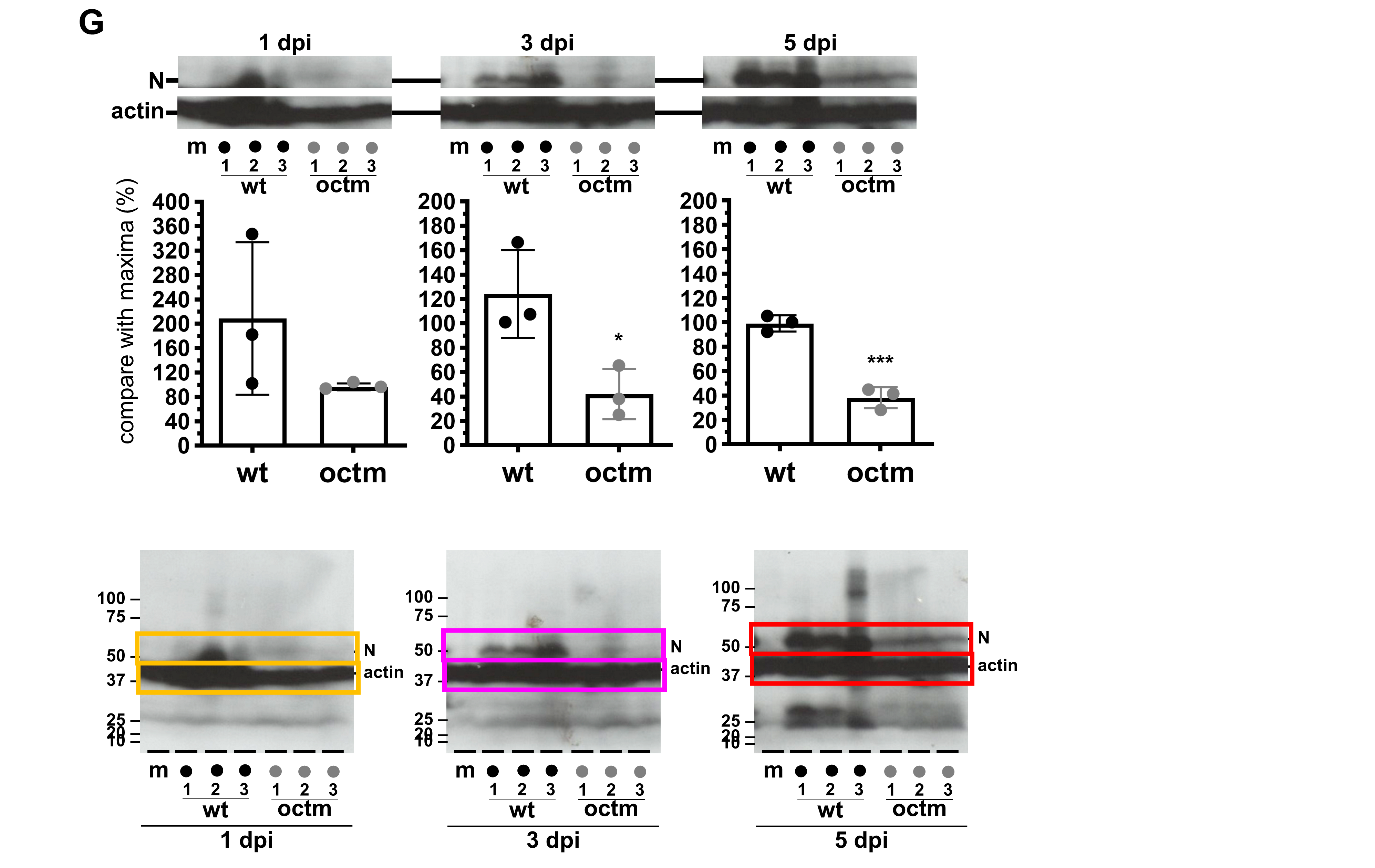


**Figure S2. Uncropped gels for Figure 5G.**


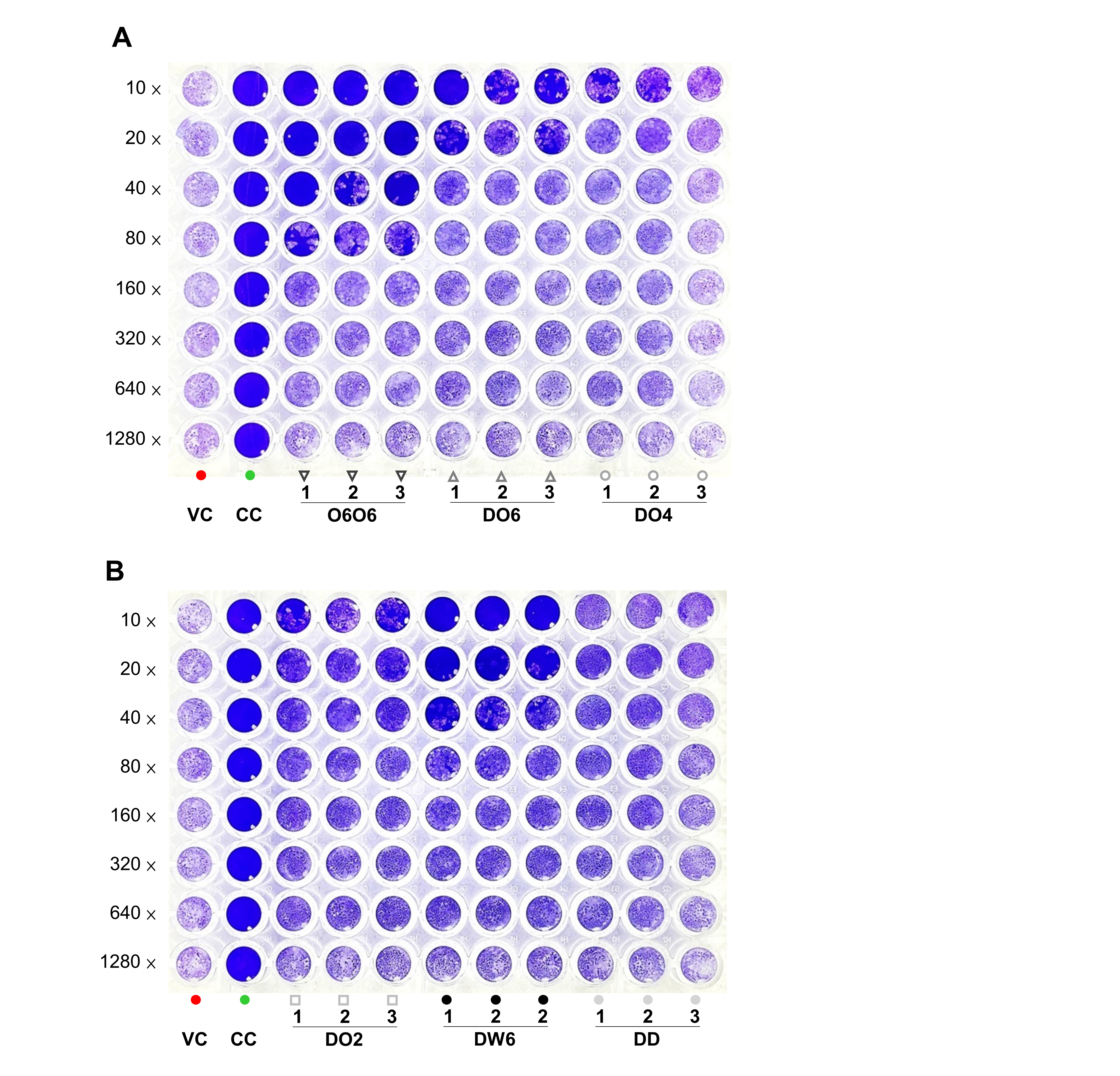


**Figure S3. Serum virus neutralization assay for groups O6O6, DO6 and DO4 (A) and groups DO2, DW6 and DD (B).** Because one mouse died in group DW6 during infection, only two samples were used for the serum virus neutralization assay. Please see the figure legend of Figure 8 for the treatments of groups DD, DO2, DO4, DO6, O6O6 and DW6. VC, virus control in which cells were infected with MHVwt; CC, cell control in which cells were mock-infected.

**Table S1. Identification of the conserved octamers derived from different genera of coronaviruses**


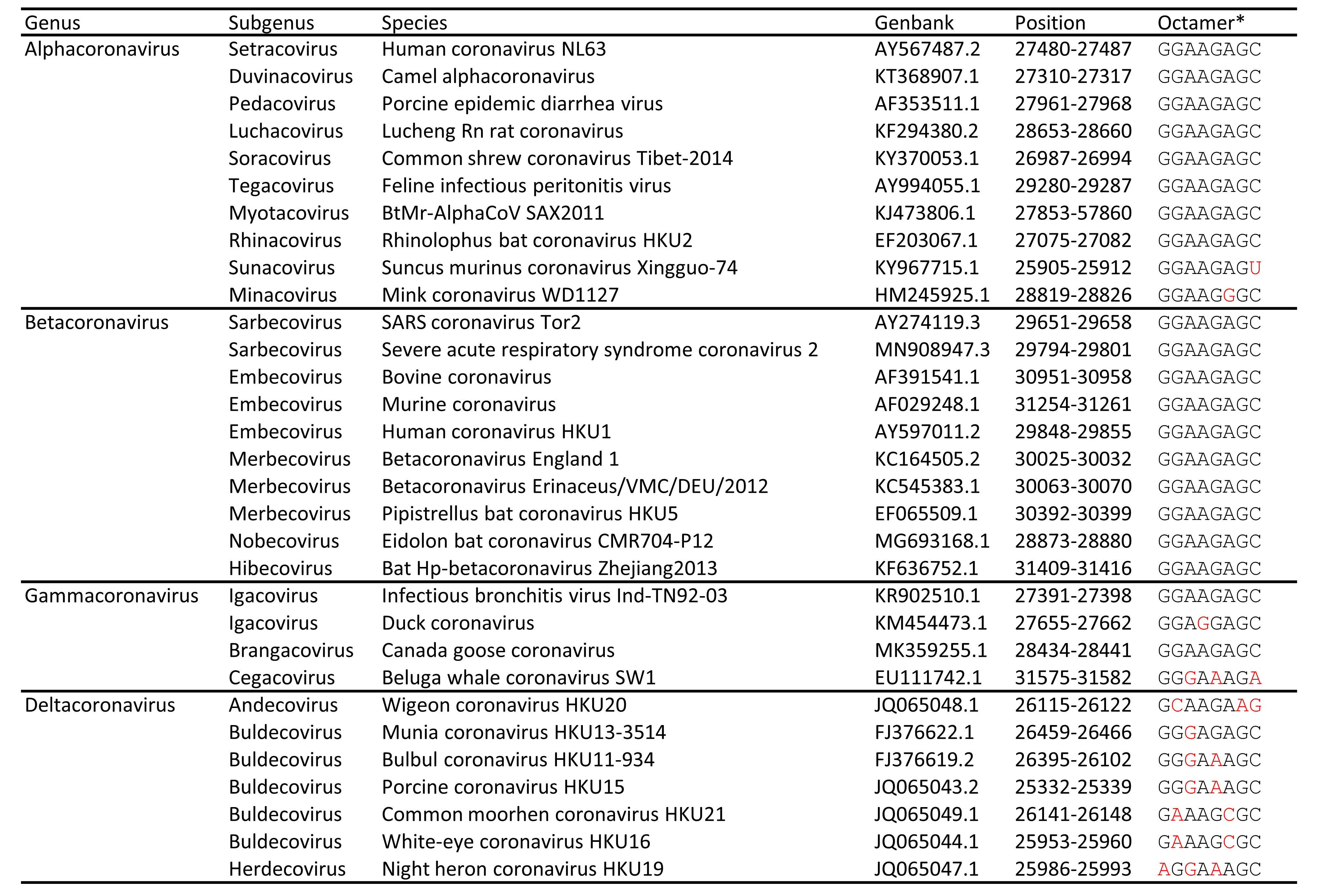


* Nucleotides that differ from those in the octamer GGAAGAGC are indicated in red.
